# Genetic Variation in the *TP53* Pathway and Bladder Cancer Risk. A Comprehensive Analysis

**DOI:** 10.1371/journal.pone.0089952

**Published:** 2014-05-12

**Authors:** Silvia Pineda, Roger L. Milne, M. Luz Calle, Nathaniel Rothman, Evangelina López de Maturana, Jesús Herranz, Manolis Kogevinas, Stephen J. Chanock, Adonina Tardón, Mirari Márquez, Lin T. Guey, Montserrat García-Closas, Josep Lloreta, Erin Baum, Anna González-Neira, Alfredo Carrato, Arcadi Navarro, Debra T. Silverman, Francisco X. Real, Núria Malats

**Affiliations:** 1 Spanish National Cancer Research Center (CNIO), Madrid, Spain; 2 Systems Biology Department, University of Vic, Vic, Spain; 3 Division of Cancer Epidemiology and Genetics, National Cancer Institute, Department of Health and Human Services, Bethesda, Maryland, United States of America; 4 Centre for Research in Environmental Epidemiology (CREAL), Barcelona, Spain; 5 Institut Municipal d'Investigació Mèdica – Hospital del Mar, Barcelona, Spain; 6 Department of Preventive Medicine, Universidad de Oviedo, Oviedo, Spain; 7 Departament de Patologia, Hospital del Mar – IMAS, Barcelona, Spain; 8 Servicio de Oncología, Hospital Universitario de Elche, Elche, Spain; 9 Servicio de Oncología, Hospital Universitario Ramon y Cajal, Madrid, Spain; 10 Departament de Ciències Experimentals i de la Salut, Universitat Pompeu Fabra, Barcelona, Spain; 11 Institut de Biologia Evolutiva (UPF-CSIC), Barcelona, Spain; 12 Institució Catalana de Recerca i Estudis Avançats (ICREA), Barcelona, Spain; 13 Instituto Nacional de Bioinformática, Barcelona, Spain; National Cancer Center, Japan

## Abstract

**Introduction:**

Germline variants in *TP63* have been consistently associated with several tumors, including bladder cancer, indicating the importance of *TP53* pathway in cancer genetic susceptibility. However, variants in other related genes, including *TP53* rs1042522 (Arg72Pro), still present controversial results. We carried out an in depth assessment of associations between common germline variants in the *TP53* pathway and bladder cancer risk.

**Material and Methods:**

We investigated 184 tagSNPs from 18 genes in 1,058 cases and 1,138 controls from the Spanish Bladder Cancer/EPICURO Study. Cases were newly-diagnosed bladder cancer patients during 1998–2001. Hospital controls were age-gender, and area matched to cases. SNPs were genotyped in blood DNA using Illumina Golden Gate and TaqMan assays. Cases were subphenotyped according to stage/grade and tumor p53 expression. We applied classical tests to assess individual SNP associations and the Least Absolute Shrinkage and Selection Operator (LASSO)-penalized logistic regression analysis to assess multiple SNPs simultaneously.

**Results:**

Based on classical analyses, SNPs in *BAK1* (1), *IGF1R* (5), *P53AIP1* (1), *PMAIP1* (2), *SERINPB5* (3), *TP63* (3), and *TP73* (1) showed significant associations at p-value≤0.05. However, no evidence of association, either with overall risk or with specific disease subtypes, was observed after correction for multiple testing (p-value≥0.8). LASSO selected the SNP rs6567355 in *SERPINB5* with 83% of reproducibility. This SNP provided an OR = 1.21, 95%CI 1.05–1.38, p-value = 0.006, and a corrected p-value = 0.5 when controlling for over-estimation.

**Discussion:**

We found no strong evidence that common variants in the *TP53* pathway are associated with bladder cancer susceptibility. Our study suggests that it is unlikely that *TP53* Arg72Pro is implicated in the UCB in white Europeans. *SERPINB5* and *TP63* variation deserve further exploration in extended studies.

## Introduction

In more developed countries, urothelial carcinoma of the bladder (UCB) is the fourth most common cancer in men and the seventeenth in women, the overall male∶female ratio being 3∶1. This ratio is greater (6∶1) in Spain, where the disease presents one of the highest incidence rates among men (51 per 100,000 man-year) [1]. Tobacco smoking and occupational exposure to aromatic amines have been established as the strongest risk factors, among others [2]. While no high-penetrance allele/gene has been identified to date as associated with UCB, there is well-established evidence that UCB risk is influenced by common genetic variants [3], [4].

Previous studies characterizing UCB are consistent with the existence of, at least, two disease subtypes based on their morphological and genetic features. The first subtype includes low-risk, papillary, non-muscle invasive tumors (NMIT, 60–65% of all UCB) and the second type includes both high-risk NMIT (15–20% of all UCB) and muscle invasive tumors (MIT, 20%–30% of all UCB). Supporting these morphological subtypes, differential genetic pathways were described and were associated with distinct UCB evolution. Somatic mutations in *FGFR3* are more frequent in low-risk NMIT, while mutations in *TP53* and *RB* are mainly involved in high-risk NMIT and MIT [5], [6]; mutations in *PIK3CA* and *HRAS* occur similarly in the two tumor subtypes. Interestingly, an exploratory analysis has shown that some germline genetic variants might be differentially associated with the risk of developing distinct UCB subphenotypes defined according to tumor stage (T) and grade (G) [7].

*TP53* is the most important human tumor suppressor gene and its implications in UCB have been extensively studied [8]. *TP53* is located in17p13, a region that is frequently deleted in human cancers, and it encodes the p53 protein. p53 is a transcription factor controlling cell proliferation, cell cycle, cell survival, and genomic integrity and - therefore - it regulates a large number of genes. Under normal cellular conditions, p53 is rapidly degraded due to the activity of *MDM2*, a negative p53 regulator that is also a p53 target gene. Upon DNA damage or other stresses, p53 is stabilized and regulates the expression of many genes involved in cell cycle arrest, apoptosis, and DNA repair among others. Somatic alterations in *TP53*/p53 are one of the most frequent alterations associated with UCB, especially with the more aggressive tumors [9].

Germline *TP53* mutations predispose to a wide spectrum of early-onset cancers and cause Li-Fraumeni and related syndromes [10], [11]. These mutations are usually single-base substitutions. Over 200 germline single nucleotide polymorphisms (SNPs) in *TP53* have been identified at present [12]. SNP rs1042522 (Arg72Pro) has been assessed in association with several cancers, among them UCB. However, the results of these studies are inconsistent [13], [14], [15], [16], [17], [18]. In contrast, an association between SNP rs710521 in *TP63*, a *TP53* family member, and risk of UCB has been convincingly replicated, pointing to the involvement of *TP53* pathway members in UCB susceptibility [4].

The aim of this study was to comprehensively investigate whether germline SNPs in genes involved in the *TP53* pathway are associated with risk of UCB. To this end, a total of 184 tagSNPs in 18 key genes were assessed using data from the Spanish Bladder Cancer/EPICURO study.

## Materials and Methods

### Study Subjects

The Spanish Bladder Cancer/EPICURO Study is a case-control study carried out in 18 hospitals from five areas in Spain and described elsewhere [2], [4], [7]. Briefly, cases were patients diagnosed with primary UCB at age 21–80 years between 1998 and 2001. All participants were of self-reported white European ancestry. Diagnostic slides from each patient were reviewed by a panel of expert pathologists to confirm the diagnosis and to ensure that uniform classification criteria were applied based on the 1999 World Health Organization and International Society of Urological Pathology systems [19].

Controls were patients admitted to participating hospitals for conditions thought to be unrelated to the UCB risk factors. The main reasons for hospital admission were: hernia (37%), other abdominal surgery (11%), fracture (23%), other orthopaedic problem (7%), hydrocoele (12%), circulatory disorder (4%), dermatological disorder (2%), ophthalmological disorder (1%), and other diseases (3%). Controls were individually matched to the cases on age within 5-year categories, gender, ethnic origin and region of residence.

Information on sociodemographics, smoking habits, occupational and environmental exposures, and past medical and familial history of cancer was collected by trained study monitors who conducted a comprehensive computer- assisted personal interview with the study participants during their hospital stay. Of 1,457 eligible cases and 1,465 controls, 1,219 (84%) and 1,271 (87%), were interviewed, respectively.

All subjects gave written informed consent to participate in the study, which was approved by the ethics committees of the participating centers.

### Genotyping

A total of 184 tagSNPs from 18 genes participating in the *TP53* pathway were selected using the Select Your SNPs (SYSNPs) program [20]. SYSNP used information from dbSNP b25, hg17 and HapMap Release #21. Haploview's Tagger algorithm (v3.32) was applied with default parameter values. The tool considers all available information for each SNP and implements algorithms that provide the status of each SNP as a tagSNP, a captured SNP or a non-captured SNP. According to this information tagSNPs were selected. The following groups of genes were considered: 1) *TP53* family members (*TP53*, *TP63* and *TP73*) and 2) genes known to be targets of p53 or regulators of p53 function [*BAK1, BAX, BBC3, BIRC5, CDKN1A, FAS, GADD45A, IGF1R, MDM2, PCNA, PMAIP1, SERPINB5, SFN* (Stratifin, 14-3-3sigma), *TP53AIP1*), and 3) c-*MYC*, a major oncogene involved in a broad range of human cancers that regulates p53 pro-apoptotic activity (See Table S1 in File S1). SNPs were genotyped using Illumina Golden Gate and TaqMan (Applied Biosystems) assays at the Spanish Core Genotyping Facility at the CNIO (CEGEN- CNIO). Genotyping was successful for 1,058 cases and 1,138 controls. We calculated the coverage for each gene using Haploview 4.2 by selecting the SNPs within a gene with a MAF≥0.05 from the 1000 genomes project, as reference, and obtained the number of SNPs captured with the SNPs genotyped at r2≥0.8 within each gene.

### Statistical Analysis

Departure from Hardy-Weinberg equilibrium was assessed in controls using Pearson's chi-squared test. Missing genotypes were imputed for the multi-SNP model using the BEAGLE 3.0 method [21]. Associations between UCB and the SNPs considered were assessed using two approaches: classical logistic and polytomous regression analyses applied to each SNP individually, and the Least Absolute Shrinkage and Selection Operator (LASSO)-penalized logistic regression to assess all SNPs simultaneously. All models were adjusted for age at diagnosis (cases) or interview (controls), gender, region, and smoking status. Smoking status was coded in four categories (never: <100 cigarettes in their lifetime; occasional: at least one per day for ≥6 months; former: if they had smoked regularly, but stopped at least 1 year before the study inclusion date; and current: if they had smoked regularly within a year of the inclusion date [2].

With the “classical” statistical approaches we assessed SNP main effects for the whole disease and for different subtypes of UCB, as well as SNP*SNP and SNP*smoking interactions. Disease subtypes were defined in two ways. First, according to established criteria based on tumor stage (T) and grade (G) as low-risk NMIT (TaG1 and TaG2), high-risk NMIT (TaG3, T1G2, T1G3, and Tis), and MIT (T2, T3, and T4); and second, according to the tumor expression of p53 determined using DO7 antibody. We applied the histoscore as 
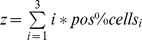
, where 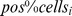 was the percentage of cells with intensity 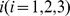. We then classified cases as having low or high p53 expression relative to the median histoscore.

To assess overall main effects, the four modes of inheritance were considered: co- dominant, dominant, recessive, and additive. The statistical significance of associations was determined using the Likelihood Ratio Test (LRT). We evaluated associations between individual SNPs and subtypes of UCB using polytomous logistic regression. Heterogeneity by disease subtype was tested by a LRT comparing this model to that with the ln(OR) restricted to be equal across subtypes. We also evaluated all two-way interactions between SNPs by a LRT comparing logistic regression models with the two SNPs (additive model) and covariates described above, with and without a single interaction term for multiplicative, per-allele effects. Interactions between each SNP and cigarette use (never vs. ever) were assessed using a similar method. Multiple testing was accounted for by applying a permutation test with 1,000 replicates. We applied Quanto (http://hydra.usc.edu/gxe/) to assess statistical power considering the available sample size.

We also assessed combined SNP effects using LASSO. The method has been described in detail by [22]. Briefly, the log-likelihood function applied in classical logistic regression
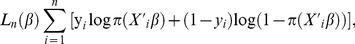
(1)where *n* is the number of observations, is reconstructed incorporating a penalty so that
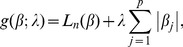
(2)where *p* is the number of SNPs and *λ* is the lasso penalty. The Newton-Raphson algorithm is applied to equation (2) to estimate *β*'s in an iterative way.

The LASSO method is based on the idea of removing irrelevant predictor variables (β = 0) via the penalty parameter, thereby selecting only the most relevant SNPs as the subset of markers most associated with the disease. The application of the penalty parameter also avoids overfitting due to both high-dimensionality and collinearity between covariates. We only considered additive genetic mode of inheritance.

This technique gives biased estimators to reduce their variance. Because of this, the implemented package in R does not provide estimates p-values for the regression beta coefficients, since standard errors are not meaningful under a biased estimator. We therefore evaluated the results by first applying the LASSO using a 5-fold cross-validation (CV) method [23] to choose the optimal λ as that giving the minimum Akaike information criterion (AIC); we then selected the subset of SNPs that were most informative with that λ. We assessed the robustness of each SNP selected in the optimal model by calculating the reproducibility as the proportion of times each SNP was selected to be in the multivariate model from 1,000 bootstrap subsamples [24].

To evaluate the association with UCB risk of that subset of SNPs, we tested them by the LRT in a multivariate regression model with all the SNPs in comparison to the null model. To correct for the over-estimation due the pre-selection of the best SNPs, we performed a permutation test with 10,000 replicates.

STATA 10 was used to run the classical logistic and multinomial regression analyses. All other statistical analyses were run in R (http://www.R-project.org), using the penalized library [25] for LASSO penalized logistic regression.

## Results

Table 1 shows the distribution of the study subjects included in the analysis: 1,058 cases and 1,138 controls. Most individuals (87%) were male and cases were more likely to be current smokers than controls (43% vs. 25%, respectively, p-value<0.001).

**Table 1:** Demographics and smoking status of patients included in the study.

	Cases (n = 1058)	Controls (n = 1138)	1 *p-value*
**Gender**			
Male	920 (87%)	991 (87%)	
Female	138 (13%)	147 (13%)	0.9
**Age**			
<55	149 (14%)	181 (16%)	
55–64	222 (21%)	278 (24%)	
65–69	241 (23%)	263 (23%)	
70–74	225 (21%)	222 (20%)	
75+	221 (21%)	194 (17%)	0.06
**Region**			
1-Barcelona	214 (20%)	233 (21%)	
2-Valles	173 (16%)	181 (16%)	
3-Elche	83 (8%)	80 (7%)	
4-Tenerife	195 (19%)	207 (18%)	
5-Asturias	393 (37%)	437 (38%)	0.9
**Smoking**			
Never	147 (14%)	334 (29%)	
Occasional	43 (4%)	81 (7%)	
Former	409 (38%)	429 (38%)	
Current	454 (43%)	283 (25%)	<0.001
Missing	5 (1%)	11 (1%)	

1 *p-value* from Pearson's χ^2^ test for association.

No evidence of departure from Hardy-Weinberg equilibrium was observed for any SNPs after consideration of multiple testing (unadjusted p-value>10^−4^). Polymorphisms in *TP53* were not individually associated with UCB risk, even at a nominal, uncorrected 5% significance level (uncorrected p-value>0.4). The percentage of reproducibility from the LASSO model using 1,000 bootstrap subsamples was <50%, indicating a poor robustness of the models. Results for the additive and co-dominant models are summarized in Table 2.

**Table 2:** SNPs in *TP53* and bladder cancer risk.

	Cases			Controls			Additive model			Co-dominant model							Repr. (%)
SNP	AA	Aa	aa	AA	Aa	aa	OR	95% CI	*p-value*	OR(Aa)	95% CI	*p-value*	OR(aa)	95% CI	*p-value*	P-trend	
rs1042522^1^	588	372	72	628	388	84	1.04	0.91–1.20	0.5	1.10	0.91–1.33	0.3	0.97	0.68–1.37	0.8	0.5	24%
rs12951053	915	109	3	972	122	5	0.98	0.75–1.27	0.9	1.04	0.79–1.39	0.7	0.64	0.14–2.88	0.5	0.8	35%
rs1625895	761	241	28	793	266	26	1.04	0.88–1.24	0.6	0.99	0.80–1.21	0.9	1.28	0.73–2.26	0.4	0.7	13%
rs2287497	835	183	9	869	207	11	0.95	0.77–1.17	0.7	0.97	0.77–1.22	0.8	0.70	0.28–1.74	0.4	0.7	48%
rs2909430	749	251	28	800	272	27	1.03	0.87–1.23	0.7	1.04	0.85–1.27	0.7	1.23	0.70–2.16	0.5	0.7	36%
rs8073498	425	467	132	435	521	128	0.99	0.87–1.13	0.9	0.94	0.78–1.13	0.5	1.01	0.75–1.34	0.9	0.8	44%
rs8079544	923	103	2	993	102	4	1.05	0.79–1.39	0.7	1.10	0.81–1.48	0.5	0.42	0.07–2.33	0.3	0.5	40%

*Repr. (%)*,percentage reproducibility assessing the robustness of each SNP by LASSO.

AA, Aa and aa represent common-homozygotes, heterozygotes and rare-allele homozygotes, respectively.

OR, odds ratio; CI, confidence interval; OR(Aa) and OR(aa) were estimated relative to genotype AA.

1 Arg72Pro polymorphism.

All models were adjusted for age, gender, region and cigarette smoking status.

**Figure 1:**
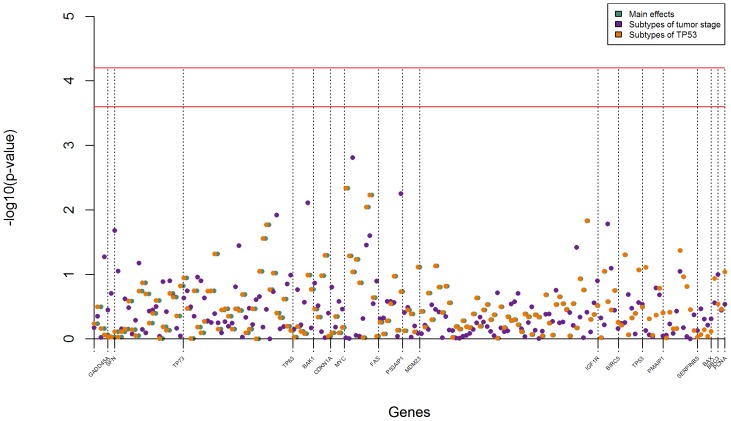
Main effect *p-values* for bladder cancer risk (overall and for each subphenotype) for each tag-SNP under the additive mode of inheritance. A SNP *p-value* above the red line is considered as associated with the phenotype after multiple testing correction by Bonferroni (4.2 for main effects and 3.6 for subtypes). All models are adjusted for age, gender, region and cigarette smoking status.

Using classical logistic regression, SNPs in *BAK1* (1), *IGF1R* (5), *P53AIP1* (1), *PMAIP1* (2), *SERPINB5* (3), *TP63* (3), and *TP73* (1) showed significant results, at a non-corrected p-value≤0.05, with overall UCB risk (Table 3). However, no evidence of association with risk was observed for any individual SNPs after correcting for multiple testing (permutation test p-value>0.8). This was also the case for the associations with the established disease subtypes defined according to stage/grade or by p53 expression (Figure 1). Of note, SNPs rs3758483 and rs983751 in *FAS* were differentially and inversely associated with MIT and high p53 expressing tumors in uncorrected analyses (Tables S2 and S3 in File S1). We also observed no evidence of SNP*SNP interactions or interactions between SNPs and smoking status (data not shown).

**Table 3:** Significant SNPs at α = 0.05 in the logistic regression main effect models.

		Cases			Controls					Risk of bladder cancer				
GENE	SNP	AA	Aa	aa	AA	Aa	Aa	MAF(a)	pHWE	OR	95% CI	*p-value*	MOI	*Repr. (%)*
*BAK1*	rs11757379	654	330	42	642	390	54	0.23	0.67	0.86	0.74–0.99	0.047	Add.	33%
*IGF1R*	rs1058696	968	56	2	998	90	0	0.04	-	0.63	0.44–0.90	0.010	Dom.	81%
*IGF1R*	rs12591122	758	244	25	824	250	14	0.13	0.34	2.23	1.11–4.51	0.025	Rec.	66%
*IGF1R*	rs4966015	722	283	21	771	276	41	0.16	0.01	0.44	0.25–0.77	0.004	Rec.	43%
*IGF1R*	rs702497	633	342	50	645	366	73	0.24	0.04	0.69	0.50–0.94	0.019	Rec.	73%
*IGF1R*	rs7166348	618	365	45	670	355	62	0.22	0.11	0.67	0.44–1.00	0.050	Rec.	33%
*P53AIP1*	rs2604235	431	484	109	463	473	149	0.36	0.11	0.74	0.56–0.97	0.029	Rec.	30%
*PMAIP1*	rs1942919	270	547	207	353	509	224	0.44	0.11	1.27	1.05–1.55	0.015	Dom.	33%
*PMAIP1*	rs7240884	449	476	100	477	471	138	0.34	0.20	0.75	0.56–0.99	0.047	Rec.	25%
*SERPINB5*	rs1509476	532	413	82	614	405	69	0.25	0.87	1.20	1.04–1.38	0.012	Add.	14%
*SERPINB5*	rs1509478	378	490	159	450	493	139	0.36	0.84	1.18	1.04–1.34	0.011	Add.	51%
*SERPINB5*	rs6567355	466	442	114	552	435	93	0.29	0.60	1.21	1.05–1.38	0.006	Add.	83%
*TP63*	rs12489753	863	159	5	934	146	7	0.07	0.65	1.31	1.02–1.69	0.035	Dom.	71%
*TP63*	rs13321831	847	172	8	927	155	6	0.08	1.00	1.36	1.06–1.73	0.014	Dom.	83%
*TP63*	rs6779677	328	476	224	347	547	194	0.43	0.42	1.29	1.04–1.61	0.022	Rec.	76%
*TP73*	rs3765731	554	385	86	544	446	96	0.29	0.77	0.85	0.71–1.00	0.050	Dom	71%

*MAF(a)*, minor allele frequency); *pHWE*, *p-value* from the Hardy Weinberg equilibrium test; *MOI*, Mode of Inheritance.

*Repr. (%)*, percentage reproducibility assessing the robustness of each SNP by LASSO.

All models are adjusted for age, gender, region and smoking status.

Odd ratio and 95%CI under the model of inheritance that provided the lowest *p-value*, and percentage reproducibility from LASSO under the additive mode of inheritance.

When all 184 SNPs were simultaneously assessed using LASSO, the method selected rs6567355 in *SERPINB5* with a reproducibility = 83%. This SNP provided an OR = 1.21, 95%CI 1.05–1.38, p-value = 0.006 in the main effect logistic regression model and a corrected p-value = 0.5 when controlling for over-estimation (Table 3). While not selected by LASSO in the last model under the stringent criteria applied, *IGF1R*-rs1058696 (OR = 0.63, 95%CI 0.44–0.90, p-value = 0.010) and *TP63*-rs13321831 (OR = 1.36, 95%CI 1.06–1.73, p-value = 0.014) showed a percentage of reproducibility >80%.

## Discussion

We genotyped common variants in genes in the *TP53* pathway in 1,058 cases and 1,138 controls of white European ancestry and found no strong evidence of association with risk of UCB overall, or with subtypes of the disease defined by stage and grade or by p53 expression.

A key gene in the pathway is *TP53*, and the most commonly studied variant in this particular gene is Arg72Pro (rs1042522). Its implication in susceptibility to various cancers has been reported in Asian populations, but not in white Europeans. A meta- analysis of 49 cervical cancer studies contributing a total of 7,946 cases and 7,888 controls found that the Arg allele was associated with an increased risk of cervix cancer [14]. However, another meta-analysis of 39 studies (26,041 cases and 29,679 controls) found weak evidence for an association of the same variant with reduced breast cancer risk [18]. Regarding gastric cancer, a combined analysis of 6,859 cases and 9,277 controls from 28 studies found a stronger inverse association only among Asians [26]. For lung cancer, a marginally significant increased risk was in a combined analysis of data with 15,647 cases and 14,391 controls from 36 studies, though the association seemed to be also confined to the Asian population [27].

The association between *TP53* Arg72Pro and UCB risk has been assessed by two meta-analyses. Overall, no association was observed by Jiang et al. when comparing 1,601 cases and 1,948 controls from 10 studies, although a marginally significant association was seen among Asians (OR = 0.77, 95%CI 0.59–1.00, for ArgArg/ArgPro vs. ProPro) [13]. Discordant results have been recently reported combining data from 14 studies contributing with 2,176 cases and 2,798 controls (OR = 1.268, 95%CI 1.003–1.602, for ArgArg/ArgPro vs. ProPro among the Asian population) [17]. A large number of studies overlap between the two meta-analyses. The lack of information on gene-gene and gene-environment interactions, as well as on the concomitant effect of *TP53* somatic mutations may explain the discordant results [28].

The findings from our study confirm the lack of association of Arg72Pro in *TP53* with risk of UCB in white Europeans (OR = 0.98, 95%CI 0.77–1.26, for ArgPro vs. ArgArg and OR = 0.91, 95%CI 0.75–1.09, for ProPro vs. ArgArg, p-value = 0.5 for overall effects) [13], [17]. However, we cannot rule out that lack of statistical power may hamper identification of a small effect association: even with its large sample size, the present study sample size could detect an OR≥1.3 per-allele for this SNP with 90% statistical power and at a significance level of 5%.

Regarding other SNPs in *TP53*, Lin et al reported an association with rs9895829 and rs1788227 (p-value = 0.003 and 0.027, respectively) in a smaller study with 201 cases and 311 controls in an Asian population [29]. We did not genotype these SNPs, though they are in high LD with two SNPs considered here: rs8079544 (LD = 1.0) and rs12951053 (LD = 0.7), respectively. Nonetheless, none of the assessed additional SNPs in *TP53* appeared to be associated with UCB risk. The partial coverage of the gene with the assessed SNPs (38%) does not allow us to dismiss the role of *TP53* in UCB susceptibility.

*TP63* is another key member of the studied pathway. One SNP (rs710521) located in this gene has been reported to be associated with risk of UCB by a GWAS (per-allele OR = 1.19, 95%CI 1.12–1.27, p-value = 1.15×10^−7^) [30]. This association was convincingly replicated in a combined analysis of data from different studies (allele-specific OR = 1.18, 95%CI 1.12–1.24, p-value = 1.8×10^−10^), including ours, for which it was genotyped as part of a separate initiative [4]. Of note, this particular SNP did not show significant results in our study (OR = 0.95, 95%CI 0.83–1.10, p-value = 0.5), a fact that can be explained by the different geographical location related exposures of the participating studies, being UCB an environmental driven disease [31]. The present study assessed 32 SNPs in *TP63*, providing 24% of the gene coverage. Three of them showed uncorrected significant results in the overall UCB association analysis with a percentage of reproducibility >70% from LASSO. These results warrant an extended UCB study on this region.

Regarding other SNPs in the selected genes, we did not find any strong evidence of association after correcting for multiple testing (permutation test p-value≥0.8 for overall main effects and p-value≥0.3 for subtype effects). The top (uncorrected) significant SNPs were located in *BAK1*, *IGF1R*, *P53AIP1*, *PMAIP1*, *SERPINB5*, and *TP73*. Common variants in these genes have not previously been reported as associated with UCB risk, though an altered expression of *BAK1* and *IGF1R* has been described in bladder tumors.

Many complex diseases, such as UCB, are likely due to the combined effects of multiple loci [32] and most traditional association studies assessing main effects for one SNP at a time are underpowered to detect small effects [33]. Therefore, the implication of common genetic variants may be better assessed by a method that both selects a far-reduced set of potentially associated SNPs and tests for association globally. This has been a challenge due to the high-dimensionality and collinearity between SNPs. Nevertheless, penalized techniques can deal with these problems and they are starting to emerge in genetic association studies. Wu et al used penalized logistic regression in a genome-wide association study applied to coeliac disease data and Zhou et al extended this work to the assessment of association for common and rare variants applied to family cancer registry data [34] [35]. In the present study, we applied the LASSO algorithm to account for the combination effects of the SNPs in the TP53 pathway and UCB risk. Under the criteria applied, this method selected one SNP (rs6567355) that showed a non-corrected p-value = 0.006 for the additive mode of inheritance with a percentage of reproducibility = 83%. This is a frequent G>A SNP (MAF = 0.29) located in the intron region of *SERPINB5*. As mentioned before, no evidences of previous association between this SNP and any disease have been reported at present. *SERPINB5* is a tumor suppressor (Table S1 in File S1). The expression levels of this gene has been correlated with those of *DBC1* (Deleted in bladder cancer 1) in UCB specimens, suggesting its involvement in the urokinase-plasminogen pathway [36]. *SERPINB5* would deserve of further exploration in extended studies, as well.

A limitation of our study is the incomplete tagging of the selected genes due to the use of an earlier HapMap release to select tag SNPs, prior to the availability of data from the 1000 genomes project. The median coverage of the 18 genes considered in the pathway is, according to the updated HapMap releases, 44%, ranging from 21% to 86%. Therefore, we cannot rule out completely the implication of common variation in these genes in UCB susceptibility.

For common SNPs (MAF>0.05), our study is powered (90%) to detect ORs≥1.4 at a significance level of 0.05, assuming an additive mode of inheritance. Therefore, the study is not conclusive with OR<1.4. While this study represents one of the largest assessments conducted till present, much larger studies will be required to rule out smaller main effects associated with common variants in the genes of this pathway. This is even more important when subphenotype analyses are considered. We also found no evidence of SNP-SNP interactions (permutation test p-value≥0.3) and SNP-smoking interactions (permutation test p-value≥0.07), although the power was even more limited to detect these. According to the candidate pathway, the studied SNPs were selected as tags; therefore, they were not correlated showing a low LD. This fact, let us overcome a potential limitation affecting the percentage of reproducibility when SNPs are high correlated.

Credit should also be given to this study, not only regarding its large sample size, but also for its prospective nature and disease representativeness, for the homogeneous methods applied to collect information and biosamples by the participating centers, for the integration of different type of information (sociodemographics, epidemiological, genetic, clinical and pathological, and molecular), and for the comprehensive and innovative statistical approaches applied to assess UCB susceptibility associated with a highly candidate pathway.

In conclusion, using a comprehensive analysis accounting different models and different approaches, we found no strong evidence that common variants in the *TP53* pathway are associated with UCB risk. However, specific members of the pathway, *TP63* and *SERPINB5* deserve of further exploration in extended studies. On the other hand, our study suggests that it is unlikely that *TP53* Arg72Pro is implicated in the UCB in white Europeans.

While biological sound, candidate pathway analysis have throw limited acknowledge in the genetic susceptibility field of many diseases. The reasons of this relative poor efficiency may be, among others, the still lack of knowledge of all key components of a given pathway, the introduction of noise by considering many genes/variants without showing association, and the lack of coverage of rare variants not tagged through this approach, in addition to methodological explanations such as an impaired statistical power. Scientists should review whether it is time to dismiss this approach towards a more comprehensive strategy such whole genome/exome sequencing in dissecting the genetic architecture of complex diseases.

## Supporting Information

File S1 Combined Supporting Information file containing: Table S1, Location and function of the selected genes. Table S2, Heterogeneity in single nucleotide polymorphism (SNP) risk estimates among bladder cancer subphenotypes defined according to stage and grade in the Spanish Bladder Cancer Study. Table S3, Heterogeneity in single nucleotide polymorphism (SNP) risk estimates among bladder cancer subphenotypes defined by p53 expression in the Spanish Bladder Cancer Study.
(DOCX)
