# Supplementary material for: Genetic Variation in the TP53 Pathway and Bladder Cancer Risk. A Comprehensive Analysis
Source: PLoS One. 2014 May 12;9(5):e89952. doi: 10.1371/journal.pone.0089952 (PMC4018346; doi:10.1371/journal.pone.0089952)
Supplement: File S1 — Combined Supporting Information file containing: Table S1, Location and function of the selected genes. Table S2, Heterogeneity in single nucleotide polymorphism (SNP) risk estimates among bladder cancer subphenotypes defined according to stage and grade in the Spanish Bladder Cancer Study. Table S3, Heterogeneity in single nucleotide polymorphism (SNP) risk estimates among bladder cancer subphenotypes defined by p53 expression in the Spanish Bladder Cancer Study. (DOCX) [file pone.0089952.s001.docx]

**Table A**. Location and function of the selected genes.

| **Gene** | **Alias** | **Location** | **Function** | **No SNPs** | **Gene coverage** |
| --- | --- | --- | --- | --- | --- |
| *GADD45A* | DDIT1  GADD45 | 1p31.2 | This gene is a member of a group of genes whose transcript levels are increased following stressful growth arrest conditions and treatment with DNA-damaging agents. The protein encoded by this gene responds to environmental stresses by mediating activation of the p38/JNK pathway via MTK1/MEKK4 kinase. The DNA damage-induced transcription of this gene is mediated by both p53-dependent and -independent mechanisms. Alternatively spliced transcript variants encoding distinct isoforms have been found for this gene.[provided by RefSeq, Dec 2010] | 4 | 28% |
| *SFN*  *Stratifin*  *14-3-3sigma* | YWHAS | 1p36.11 | Adapter protein implicated in the regulation of a large spectrum of both general and specialized signaling  pathways. Binds to a large number of partners, usually by recognition of a phosphoserine or phosphothreonine motif. Binding generally results in the modulation of the activity of the binding partner. When bound to KRT17, regulates  protein synthesis and epithelial cell growth by stimulating Akt/mTOR pathway (By similarity). p53-regulated inhibitor of G2/M progresión. | 1 | 64% |
| *TP73* | --- | 1p36.3 | This gene encodes a member of the p53 family of transcription factors involved in cellular responses to stress and development. It maps to a region on chromosome 1p36 that is frequently deleted in neuroblastoma and other tumors, and thought to contain multiple tumor suppressor genes. The demonstration that this gene is monoallelically expressed (likely from the maternal allele), supports the notion that it is a candidate gene for neuroblastoma. Many transcript variants resulting from alternative splicing and/or use of alternate promoters have been found for this gene, but the biological validity and the full-length nature of some variants have not been determined. [provided by RefSeq, Feb 2011] | 21 | 44% |
| *TP63* | AIS  KET  LMS  NBP  RHS  p40  p51  p63  EEC3  OFC8  p73H  p73L  SHFM4  TP53L  TP73L  p53CP  TP53CP  B(p51A)  B(p51B) | 3q28 | This gene encodes a member of the p53 family of transcription factors. An animal model, p63 -/- mice, has been useful in defining the role this protein plays in the development and maintenance of stratified epithelial tissues. p63 -/- mice have several developmental defects which include the lack of limbs and other tissues, such as teeth and mammary glands, which develop as a result of interactions between mesenchyme and epithelium. Mutations in this gene are associated with ectodermal dysplasia, and cleft lip/palate syndrome 3 (EEC3); split-hand/foot malformation 4 (SHFM4); ankyloblepharon-ectodermal defects-cleft lip/palate; ADULT syndrome (acro-dermato-ungual-lacrimal-tooth); limb-mammary syndrome; Rap-Hodgkin syndrome (RHS); and orofacial cleft 8. Both alternative splicing and the use of alternative promoters results in multiple transcript variants encoding different proteins. Many transcripts encoding different proteins have been reported but the biological validity and the full-length nature of these variants have not been determined. [provided by RefSeq, Jul 2008] | 32 | 24% |
| *CDKN1A* | P21  CIP1  SDI1  WAF1  CAP20  CDKN1  MDA-6  p21CIP1 | 6p21.2 | This gene encodes a potent cyclin-dependent kinase inhibitor. The encoded protein binds to and inhibits the activity of cyclin-CDK2 or -CDK4 complexes, and thus functions as a regulator of cell cycle progression at G1. The expression of this gene is tightly controlled by the tumor suppressor protein p53, through which this protein mediates the p53-dependent cell cycle G1 phase arrest in response to a variety of stress stimuli. This protein can interact with proliferating cell nuclear antigen (PCNA), a DNA polymerase accessory factor, and plays a regulatory role in S phase DNA replication and DNA damage repair. This protein was reported to be specifically cleaved by CASP3-like caspases, which thus leads to a dramatic activation of CDK2, and may be instrumental in the execution of apoptosis following caspase activation. Multiple alternatively spliced variants have been found for this gene. [provided by RefSeq, Nov 2010] | 5 | 34% |
| *BAK1* | BAK  CDN1  BCL2L7  BAK-LIKE | 6p21.3 | The protein encoded by this gene belongs to the BCL2 protein family. BCL2 family members form oligomers or heterodimers and act as anti- or pro-apoptotic regulators that are involved in a wide variety of cellular activities. This protein localizes to mitochondria, and functions to induce apoptosis. It interacts with and accelerates the opening of the mitochondrial voltage-dependent anion channel, which leads to a loss in membrane potential and the release of cytochrome c. This protein also interacts with the tumor suppressor P53 after exposure to cell stress. [provided by RefSeq, Jul 2008] | 6 | 68% |
| *MYC* | MRTL  MYCC  c-Myc  bHLHe39 | 8q24.21 | The protein encoded by this gene is a multifunctional, nuclear phosphoprotein that plays a role in cell cycle progression, apoptosis and cellular transformation. It functions as a transcription factor that regulates transcription of specific target genes. Mutations, overexpression, rearrangement and translocation of this gene have been associated with a variety of hematopoietic tumors, leukemias and lymphomas, including Burkitt lymphoma. There is evidence to show that alternative translation initiations from an upstream, in-frame non-AUG (CUG) and a downstream AUG start site result in the production of two isoforms with distinct N-termini. The synthesis of non-AUG initiated protein is suppressed in Burkitt's lymphomas, suggesting its importance in the normal function of this gene. [provided by RefSeq, Jul 2008] | 4 | 21% |
| *FAS* | APT1  CD95  FAS1  APO-1  FASTM  ALPS1A  TNFRSF6 | 10q24.1 | The protein encoded by this gene is a member of the TNF-receptor superfamily. This receptor contains a death domain. It has been shown to play a central role in the physiological regulation of programmed cell death, and has been implicated in the pathogenesis of various malignancies and diseases of the immune system. The interaction of this receptor with its ligand allows the formation of a death-inducing signaling complex that includes Fas-associated death domain protein (FADD), caspase 8, and caspase 10. The autoproteolytic processing of the caspases in the complex triggers a downstream caspase cascade, and leads to apoptosis. This receptor has been also shown to activate NF-kappaB, MAPK3/ERK1, and MAPK8/JNK, and is found to be involved in transducing the proliferating signals in normal diploid fibroblast and T cells. Several alternatively spliced transcript variants have been described, some of which are candidates for nonsense-mediated mRNA decay (NMD). The isoforms lacking the transmembrane domain may negatively regulate the apoptosis mediated by the full length isoform. [provided by RefSeq, Mar 2011] | 10 | 72% |
| *TP53AIP1* | P53AIP1 | 11q24 | This gene is specifically expressed in the thymus, and encodes a protein that is localized to the mitochondrion. The expression of this gene is inducible by p53, and it is thought to play an important role in mediating p53-dependent apoptosis. Alternatively spliced transcript variants encoding different isoforms have been described for this gene. [provided by RefSeq, Oct 2011] | 7 | 50% |
| *MDM2* | HDMX  hdm2  ACTFS | 12q14.3-q15 | This gene encodes a nuclear-localized E3 ubiquitin ligase. The encoded protein can promote tumor formation by targeting tumor suppressor proteins, such as p53, for proteasomal degradation. This gene is itself transcriptionally-regulated by p53. Overexpression or amplification of this locus is detected in a variety of different cancers. There is a pseudogene for this gene on chromosome 2. Alternative splicing results in a multitude of transcript variants, many of which may be expressed only in tumor cells. [provided by RefSeq, Jun 2013] | 5 | 69% |
| *IGF1R* | IGFR  CD221  IGFIR  JTK13 | 15q26.3 | This receptor binds insulin-like growth factor with a high affinity. It has tyrosine kinase activity. The insulin-like growth factor I receptor plays a critical role in transformation events. Cleavage of the precursor generates alpha and beta subunits. It is highly overexpressed in most malignant tissues where it functions as an anti-apoptotic agent by enhancing cell survival. [provided by RefSeq, Jul 2008] | 52 | 25% |
| *TP53* | P53  BCC7  LFS1  TRP53 | 17p13.1 | This gene encodes a tumor suppressor protein containing transcriptional activation, DNA binding, and oligomerization domains. The encoded protein responds to diverse cellular stresses to regulate expression of target genes, thereby inducing cell cycle arrest, apoptosis, senescence, DNA repair, or changes in metabolism. Mutations in this gene are associated with a variety of human cancers, including hereditary cancers such as Li-Fraumeni syndrome. Alternative splicing of this gene and the use of alternate promoters result in multiple transcript variants and isoforms. Additional isoforms have also been shown to result from the use of alternate translation initiation codons (PMIDs: 12032546, 20937277). [provided by RefSeq, Feb 2013] | 7 | 38% |
| *BIRC5* | API4  EPR-1  Survivin | 17q25 | This gene is a member of the inhibitor of apoptosis (IAP) gene family, which encode negative regulatory proteins that prevent apoptotic cell death. IAP family members usually contain multiple baculovirus IAP repeat (BIR) domains, but this gene encodes proteins with only a single BIR domain. The encoded proteins also lack a C-terminus RING finger domain. Gene expression is high during fetal development and in most tumors, yet low in adult tissues. Alternatively spliced transcript variants encoding distinct isoforms have been found for this gene. [provided by RefSeq, Jun 2011] | 6 | 51% |
| *PMAIP1* | APR  NOXA | 18q21.32 | Promotes activation of caspases and apoptosis. Promotes mitochondrial membrane changes and efflux of apoptogenic proteins from the mitochondria. Contributes to p53/TP53-dependent apoptosis after radiation exposure. Promotes proteasomal degradation of MCL1. Competes with BAK1 for binding to MCL1 and can displace BAK1 from its binding site on MCL1 (By similarity). Competes with BIM/BCL2L11 for binding to MCL1 and can displace BIM/BCL2L11 from its binding site on MCL1 | 6 | 39% |
| *SERPINB5* | PI5  Maspin | 18q21.33 | Tumor suppressor. It blocks the growth, invasion, and metastatic properties of mammary tumors. As it does not undergo the S (stressed) to R (relaxed) conformational transition characteristic of active serpins, it exhibits no serine protease inhibitory activity | 10 | 83% |
| *BBC3* | JFY1  PUMA  JFY-1 | 19q13.3-q13.4 | This gene encodes a member of the BCL-2 family of proteins. This family member belongs to the BH3-only pro-apoptotic subclass. The protein cooperates with direct activator proteins to induce mitochondrial outer membrane permeabilization and apoptosis. It can bind to anti-apoptotic Bcl-2 family members to induce mitochondrial dysfunction and caspase activation. Because of its pro-apoptotic role, this gene is a potential drug target for cancer therapy and for tissue injury. Alternative splicing results in multiple transcript variants. [provided by RefSeq, Dec 2011] | 1 | 22% |
| *BAX* | BCL2L4 | 19q13.3-q13.4 | The protein encoded by this gene belongs to the BCL2 protein family. BCL2 family members form hetero- or homodimers and act as anti- or pro-apoptotic regulators that are involved in a wide variety of cellular activities. This protein forms a heterodimer with BCL2, and functions as an apoptotic activator. This protein is reported to interact with, and increase the opening of, the mitochondrial voltage-dependent anion channel (VDAC), which leads to the loss in membrane potential and the release of cytochrome c. The expression of this gene is regulated by the tumor suppressor P53 and has been shown to be involved in P53-mediated apoptosis. Multiple alternatively spliced transcript variants, which encode different isoforms, have been reported for this gene. [provided by RefSeq, Jul 2008] | 4 | 33% |
| *PCNA* |  | 20pter-p12 | The protein encoded by this gene is found in the nucleus and is a cofactor of DNA polymerase delta. The encoded protein acts as a homotrimer and helps increase the processivity of leading strand synthesis during DNA replication. In response to DNA damage, this protein is ubiquitinated and is involved in the RAD6-dependent DNA repair pathway. Two transcript variants encoding the same protein have been found for this gene. Pseudogenes of this gene have been described on chromosome 4 and on the X chromosome. [provided by RefSeq, Jul 2008] | 3 | 86% |

**Table B**. Heterogeneity in single nucleotide polymorphism (SNP) risk estimates among bladder cancer subphenotypes defined according to stage and grade in the Spanish Bladder Cancer Study.

|  |  | **Cases** | | | **Controls** | | | | | **Risk of bladder cancer** | | | | | | | | |
| --- | --- | --- | --- | --- | --- | --- | --- | --- | --- | --- | --- | --- | --- | --- | --- | --- | --- | --- |
| **GENE** | **SNP** | **AA** | **Aa** | **aa** | **AA** | **Aa** | **Aa** | **MAF(a)** | **pHWE** | **OR (Cont./AA)** | **95% CI** | **OR**  **(Cont./Aa)** | **95% CI** | **OR**  **(Cont./aa)** | **95% CI** | ***p-value*** | | **MOI** |
| *BIRC5* | rs4789551 | 932 | 92 | 4 | 965 | 119 | 3 | 0.06 | 1.00 | 1.07 | 0.79-1.46 | 0.77 | 0.47-1.29 | 0.35 | 0.17-0.69 | 0.001 | Add. | |
| *CDKN1A* | rs3176352 | 476 | 429 | 121 | 489 | 489 | 106 | 0.32 | 0.33 | 1.07 | 0.92-1.25 | 0.81 | 0.64-1.03 | 1.16 | 0.94-1.44 | 0.03 | Add. | |
| *FAS* | rs3758483 | 802 | 215 | 10 | 833 | 242 | 11 | 0.12 | 0.20 | 1.10 | 0.86-1.39 | 0.85 | 0.59-1.24 | 0.64 | 0.43-0.94 | 0.02 | Dom. | |
| *FAS* | rs983751 | 800 | 215 | 10 | 833 | 242 | 12 | 0.12 | 0.26 | 1.09 | 0.86-1.38 | 0.85 | 0.59-1.23 | 0.65 | 0.44-0.96 | 0.03 | Dom. | |
| *IGF1R* | rs2016347 | 319 | 493 | 215 | 338 | 499 | 248 | 0.46 | 0.01 | 0.88 | 0.77-1.02 | 1.18 | 0.96-1.46 | 0.98 | 0.80-1.20 | 0.04 | Add. | |
| *IGF1R* | rs2871865 | 738 | 260 | 28 | 784 | 274 | 30 | 0.15 | 0.29 | 0.89 | 0.73-1.09 | 1.44 | 1.10-1.87 | 0.92 | 0.96-1.23 | 0.006 | Add. | |
| *P53AIP1* | rs2846690 | 638 | 341 | 48 | 666 | 347 | 72 | 0.23 | 0.01 | 0.98 | 0.79-1.20 | 0.72 | 0.52-0.99 | 1.20 | 0.89-1.61 | 0.04 | Dom. | |
| *P53AIP1* | rs3740831 | 690 | 307 | 30 | 714 | 333 | 41 | 0.19 | 0.77 | 0.87 | 0.71-1.05 | 0.72 | 0.53-0.97 | 1.19 | 0.92-1.53 | 0.02 | Add. | |
| *P53AIP1* | rs4937395 | 531 | 427 | 68 | 534 | 455 | 97 | 0.30 | 1.00 | 0.85 | 0.69-1.04 | 0.71 | 0.52-0.96 | 1.15 | 0.86-1.54 | 0.04 | Dom. | |
| *PMAIP1* | rs2023022 | 758 | 247 | 20 | 796 | 268 | 23 | 0.14 | 0.90 | 1.10 | 0.90-1.35 | 0.64 | 0.45-0.92 | 0.95 | 0.71-1.29 | 0.01 | Add. | |
| *SERPINB5* | rs17071220 | 536 | 402 | 89 | 598 | 423 | 65 | 0.25 | 0.42 | 0.90 | 0.73-1.11 | 1.54 | 1.14-2.09 | 1.15 | 0.86-1.54 | 0.004 | Dom. | |
| *TP73* | rs3765692 | 548 | 403 | 76 | 527 | 483 | 76 | 0.29 | 0.02 | 0.90 | 0.74-1.11 | 0.59 | 0.44-0.81 | 0.79 | 0.59-1.06 | 0.04 | Dom. | |
| *TP73* | rs3765695 | 684 | 303 | 41 | 692 | 368 | 28 | 0.19 | 0.01 | 0.88 | 0.73-1.07 | 0.77 | 0.57-1.03 | 1.23 | 0.95-1.58 | 0.02 | Add. | |
| *TP73* | rs4648553 | 593 | 374 | 58 | 642 | 383 | 63 | 0.23 | 0.55 | 1.21 | 0.99-1.49 | 0.93 | 0.69-1.27 | 0.81 | 0.60-1.10 | 0.03 | Dom. | |
| *MAF(a)*, minor allele frequency); *pHWE*, *p-value* from the Hardy Weinberg equilibrium test; *MOI*, Mode of Inheritance  All models are adjusted for age, gender, region and smoking status | | | | | | | | | | | | | | | | | | |

**Table C**. Heterogeneity in single nucleotide polymorphism (SNP) risk estimates among bladder cancer subphenotypes defined by p53 expression in the Spanish Bladder Cancer Study.

|  |  | **Cases** | | | **Controls** | | | | | **Risk of bladder cancer** | | | | | | |
| --- | --- | --- | --- | --- | --- | --- | --- | --- | --- | --- | --- | --- | --- | --- | --- | --- |
| **GENE** | **SNP** | **AA** | **Aa** | **aa** | **AA** | **Aa** | **Aa** | **MAF(a)** | **pHWE** | **OR (Cont./AA)** | **95% CI** | **OR**  **(Cont./Aa)** | **95% CI** | ***p-value*** | | **MOI** |
| *BAK1* | rs210137 | 588 | 385 | 52 | 643 | 382 | 64 | 0.23 | 0.50 | 1.22 | 0.98-1.51 | 0.94 | 0.76-1.16 | 0.04 | Dom. | |
| *birc5* | rs2856271 | 418 | 488 | 121 | 479 | 477 | 130 | 0.34 | 0.50 | 1.18 | 0.86-1.61 | 0.79 | 0.56-1.10 | 0.04 | Rec. | |
| *birc5* | rs3764384 | 440 | 476 | 107 | 515 | 455 | 118 | 0.32 | 0.26 | 1.19 | 0.86-1.65 | 0.74 | 0.51-1.06 | 0.02 | Rec. | |
| *birc5* | rs744120 | 572 | 389 | 66 | 606 | 411 | 68 | 0.25 | 0.94 | 0.86 | 0.72-1.02 | 1.14 | 0.97-1.35 | 0.004 | Add. | |
| *fas* | rs3758483 | 802 | 215 | 10 | 833 | 242 | 11 | 0.12 | 0.20 | 1.14 | 0.90-1.46 | 0.74 | 0.57-0.96 | 0.004 | Dom. | |
| *fas* | rs983751 | 800 | 215 | 10 | 833 | 242 | 12 | 0.12 | 0.26 | 1.13 | 0.88-1.44 | 0.75 | 0.58-0.98 | 0.007 | Dom. | |
| *gadd45a* | rs520820 | 596 | 375 | 56 | 667 | 357 | 64 | 0.22 | 0.08 | 1.02 | 0.82-1.26 | 1.31 | 1.06-1.61 | 0.04 | Dom. | |
| *gadd45a* | rs532446 | 478 | 446 | 102 | 493 | 476 | 115 | 0.33 | 1.00 | 0.80 | 0.65-0.99 | 1.14 | 0.93-1.41 | 0.005 | Dom. | |
| *igf1r* | rs11247380 | 464 | 457 | 107 | 513 | 461 | 111 | 0.31 | 0.62 | 1.25 | 1.01-1.55 | 0.98 | 0.79-1.21 | 0.049 | Dom. | |
| *igf1r* | rs2684811 | 643 | 332 | 50 | 705 | 336 | 47 | 0.20 | 0.39 | 1.25 | 1.00-1.55 | 0.95 | 0.76-1.18 | 0.03 | Dom. | |
| *igf1r* | rs8032477 | 357 | 503 | 166 | 399 | 514 | 174 | 0.40 | 0.70 | 0.84 | 0.62-1.14 | 0.19 | 0.90-1.56 | 0.04 | Rec. | |
| *myc* | rs4645943 | 945 | 78 | 1 | 998 | 85 | 4 | 0.04 | 0.13 | 1.17 | 0.83-1.66 | 0.67 | 0.45-1.02 | 0.01 | Add. | |
| *pmaip1* | rs11663656 | 658 | 331 | 38 | 666 | 375 | 46 | 0.21 | 0.53 | 0.76 | 0.59-0.98 | 1.10 | 0.88-1.39 | 0.008 | Dom. | |
| *pmaip1* | rs4558496 | 620 | 360 | 47 | 616 | 404 | 66 | 0.25 | 1.00 | 0.97 | 0.78-1.20 | 0.75 | 0.60-0.93 | 0.04 | Dom. | |
| *pmaip1* | rs7240884 | 449 | 476 | 100 | 477 | 471 | 138 | 0.34 | 0.20 | 0.84 | 0.71-0.98 | 1.05 | 0.89-1.22 | 0.02 | Add. | |
| *tp63* | rs11715710 | 503 | 431 | 90 | 551 | 459 | 76 | 0.28 | 0.15 | 0.94 | 0.62-1.44 | 1.64 | 1.15-2.35 | 0.01 | Rec. | |
| *tp63* | rs12107036 | 270 | 511 | 247 | 310 | 533 | 239 | 0.47 | 0.76 | 1.34 | 1.05-1.71 | 0.89 | 0.69-1.16 | 0.006 | Rec. | |
| *tp63* | rs6797409 | 918 | 106 | 3 | 980 | 103 | 3 | 0.05 | 0.75 | 0.85 | 0.58-1.23 | 1.32 | 0.95-1.83 | 0.03 | Dom. | |
| *tp63* | rs7612589 | 759 | 254 | 15 | 823 | 251 | 13 | 0.13 | 0.27 | 2.20 | 1.01-4.79 | 0.31 | 0.07-1.37 | 0.002 | Rec. | |
| *tp63* | rs7624324 | 489 | 435 | 100 | 547 | 450 | 86 | 0.29 | 0.66 | 1.56 | 1.10-2.21 | 0.96 | 0.65-1.41 | 0.02 | Rec. | |
| *tp73* | rs3765753 | 504 | 422 | 101 | 487 | 492 | 107 | 0.33 | 0.30 | 1.25 | 0.89-1.75 | 0.80 | 0.55-1.15 | 0.03 | Rec. | |
| *MAF(a)*, minor allele frequency); *pHWE*, *p-value* from the Hardy Weinberg equilibrium test; *MOI*, Mode of Inheritance  All models are adjusted for age, gender, region and smoking status | | | | | | | | | | | | | | | | |
